# Supplementary material for: Quantitative analysis of the optogenetic excitability of CA1 neurons
Source: Front Comput Neurosci. 2023 Aug 15;17:1229715. doi: 10.3389/fncom.2023.1229715 (PMC10465168; doi:10.3389/fncom.2023.1229715)
Supplement: Supplementary file 1 [file Presentation_1.pdf]

Supplementary Material

1 FIGURES

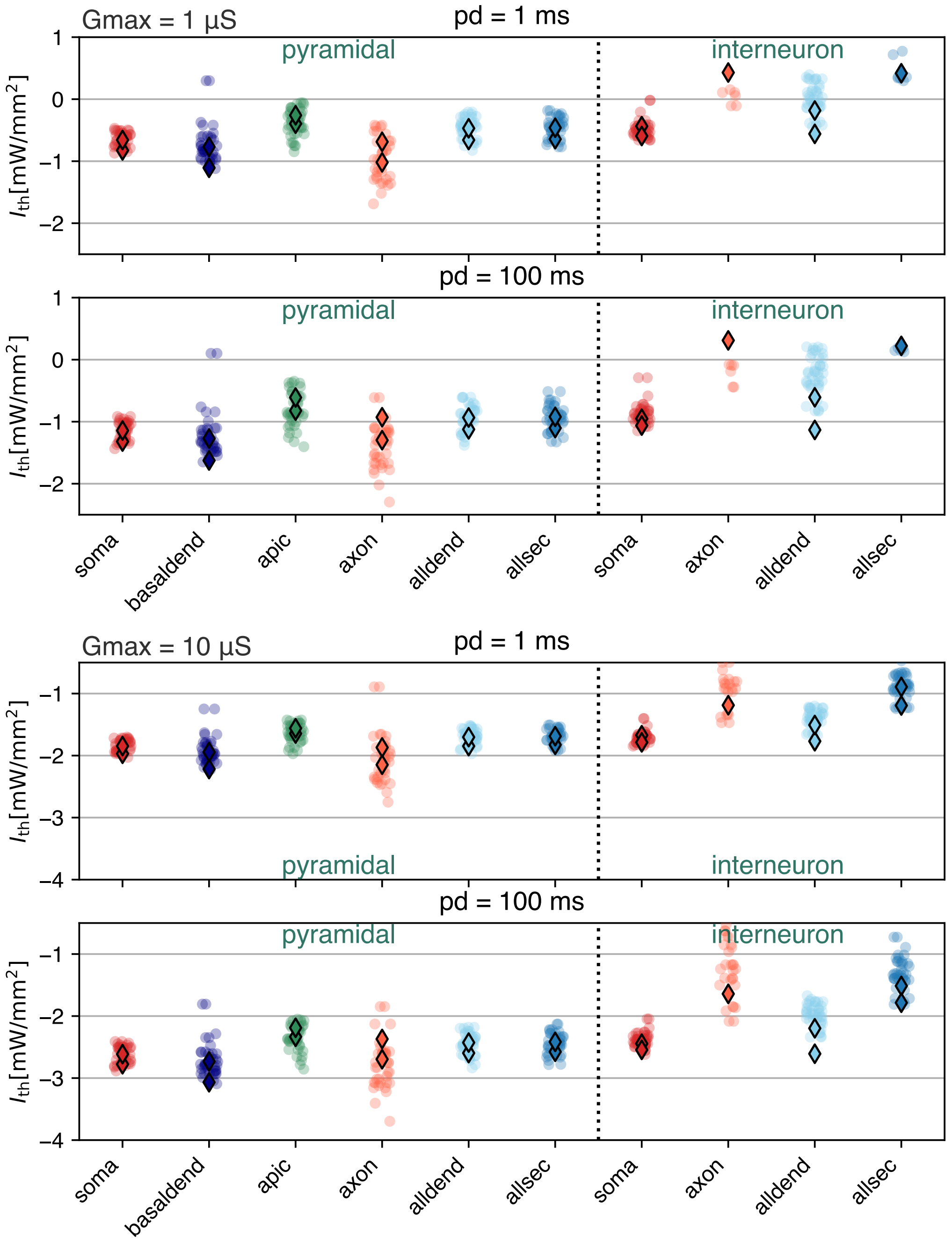

Figure S1. The threshold intensity (I_{th}) required for optogenetic excitation under uniform light stimulation of all pyramidal and continuous non-accommodating interneuron models of Migliore et al. (2018), along with virtual clones optimized using HippoUnit (Saray et al., 2021). The diamonds with a black outline represent the thresholds of the models analyzed in the paper. The titles of each subplot indicate the pulse duration (pd) and the total opsin conductance (G_{max}). The opsin is uniformly distributed over the subcellular regions, indicated on the x-axis. The model files are available at *https://wiki.ebrains.eu/bin/view/Collabs/live-paper-2021-saray-et-al/Live%20Paper*.

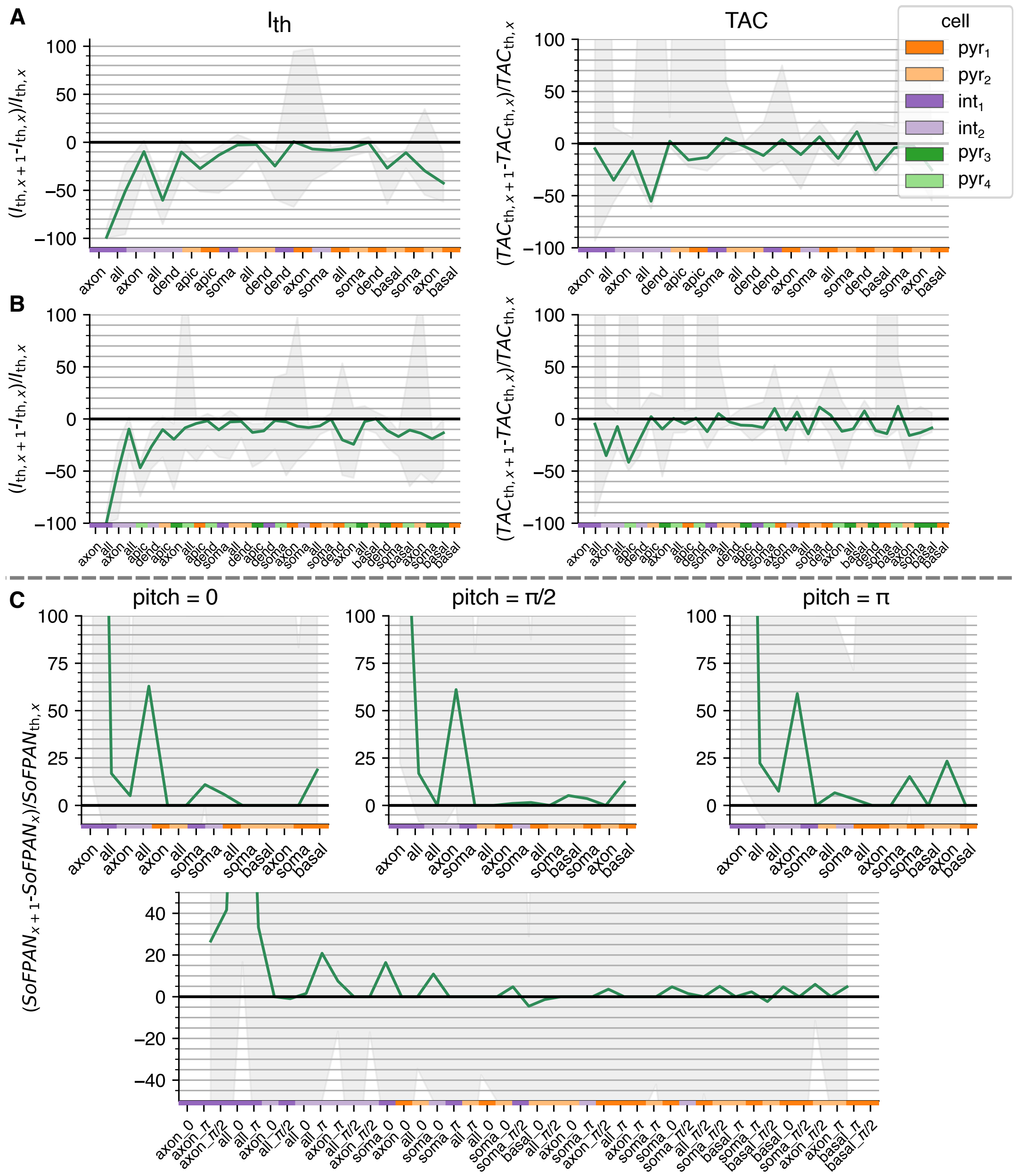

Figure S2. Median of relative change of ranked metrics. **A, B** Ranked I_{th} and TAC of the original four tested CA1 cells and with additional pyramidal cells, respectively, under a uniform intensity field. **C** Ranked SoFPAN: pitch specific at the top, all combined bottom. The gray shading indicates the total variation due to the pulse duration and opsin conductancy in all subplots. Values are same as the first offset anti-diagonal of figure S3

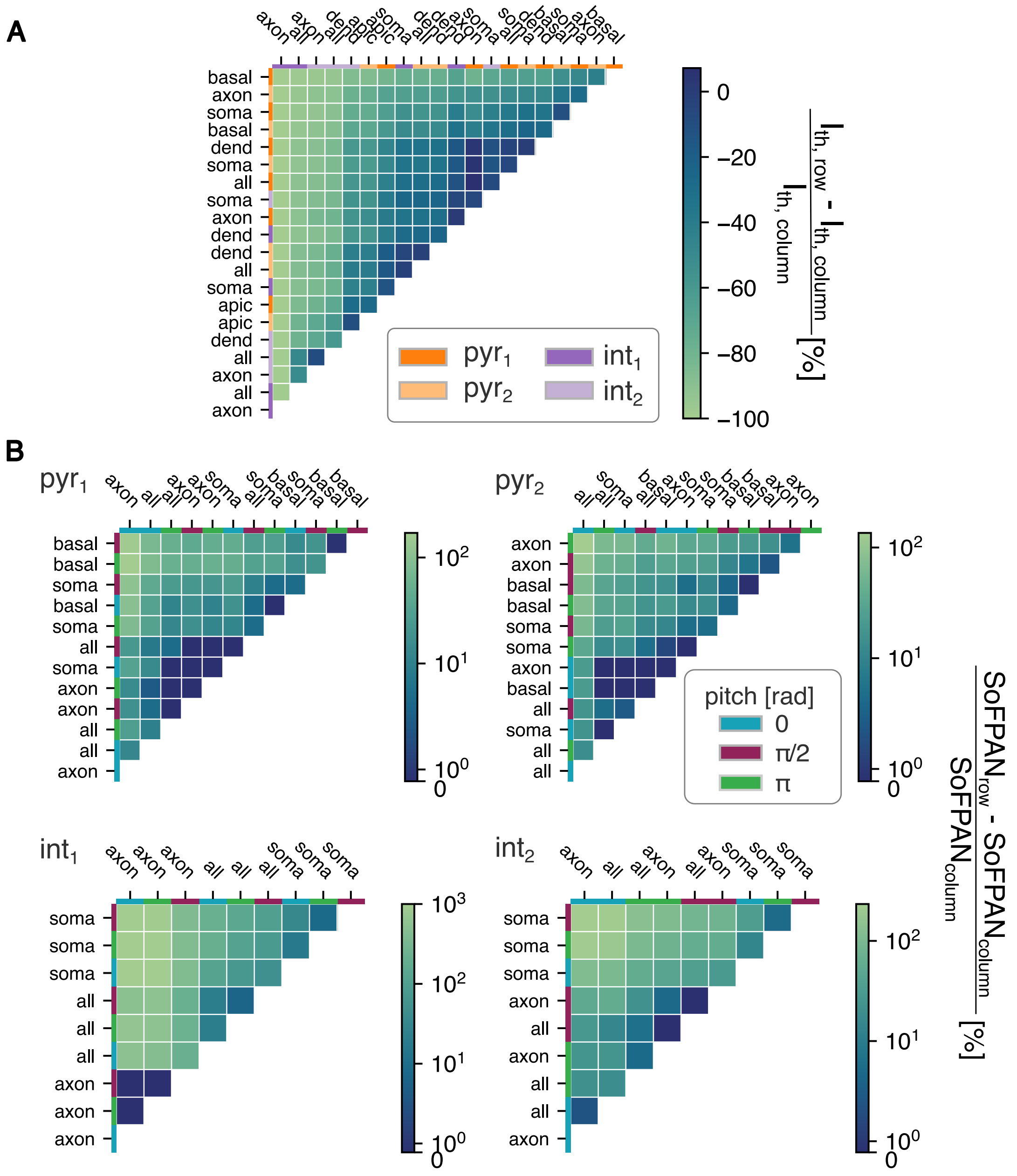

Figure S3. Median of relative differences between row and column classes with respect to column. **A** The median of relative differences in optogenetic excitation thresholds (I_{th}) under a uniform intensity field for different cell-location combinations. The values shown are the median of the relative differences calculated over all 153 combinations of pulse durations (pd) and total opsin conductance (G_{max}). **B** The median of relative differences in SoFPAN under a Monte Carlo simulated light field for different combinations of opsin locations and pitches. A separate plot is given for each of the examined cells. Here, the values are the median calculated over all the 360 pd, G_{max} and intensity at the fiber surface (I_{fiber}) combinations. The classes are sorted based on their excitability score (see manuscript section 2.4.2). The color code indicates the cell or optical fiber pitch in **A and B**, respectively.

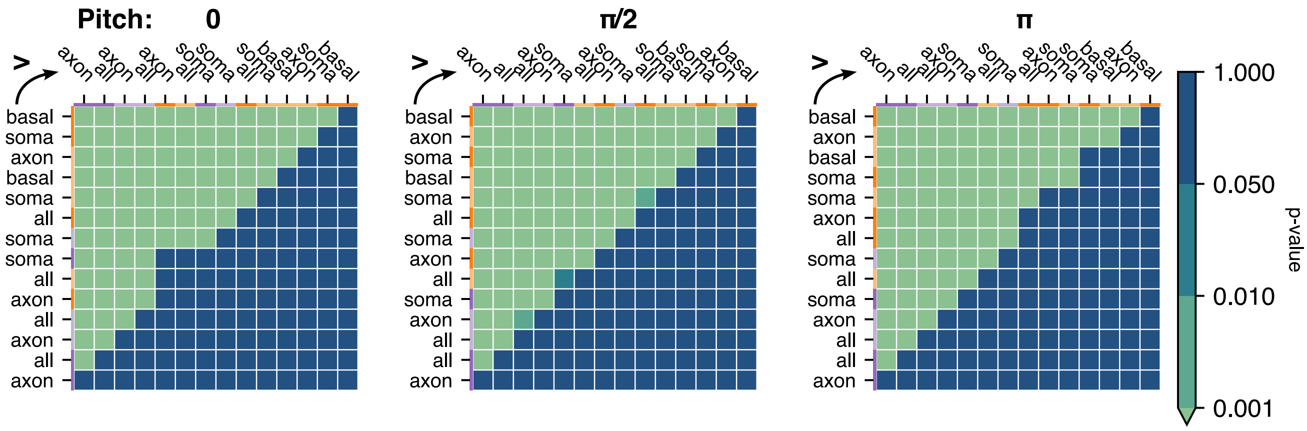

Figure S4. The p-values of the mutually, paired Wilcoxon signed-rank tests for SoFPAN comparison at a given fiber pitch. Single-sided test if class at row has higher SoFPAN than class in the column. The color code at the top and left indicates the cell model. Each population comprises 360 points: 5 pd × 8 G_{max} × 9 I_{fiber} values.

REFERENCES

Migliore, R., Lupascu, C. A., Bologna, L. L., Romani, A., Courcol, J.-D., Antonel, S., et al. (2018). The physiological variability of channel density in hippocampal ca1 pyramidal cells and interneurons explored using a unified data-driven modeling workflow. *PLOS Computational Biology* 14, e1006423. doi:10.1371/journal.pcbi.1006423

Saray, S., Rossert, C. A., Appukuttan, S., Migliore, R., Vitale, P., Lupascu, C. A., et al. (2021). *HippoUnit: A software tool for the automated testing and systematic comparison of detailed models of hippocampal neurons based on electrophysiological data*, vol. 17. doi:10.1371/JOURNAL.PCBI.1008114
